# Natural Powerhouse Duo: Hierarchical Levan Hydrogels with Nanoencapsulated Cannabidiol as Local Delivery Systems

**DOI:** 10.1007/s11095-025-03935-y

**Published:** 2025-11-11

**Authors:** Diana Solovyov, Natalia N. Porfiryeva, Rania Awad, Selay Tornaci, Maya Davidovich-Pinhas, Girts Salms, Arita Dubnika, Ebru Toksoy Öner, Alejandro Sosnik

**Affiliations:** 1 Faculty of Materials Science and Engineering, Technion Israel Institute of Technology, Haifa, 3200003 Israel; 2 Department of Bioengineering, IBSB, Marmara University RTE Campus, Istanbul, Turkey; 3 Laboratory of Food Materials Engineering, Department of Biotechnology and Food Engineering, Technion – Israel Institute of Technology, Technion City, Haifa, 3200003 Israel; 4 Biomaterials Centre of Excellence, Headquarters at Riga Technical University, Riga, Latvia; 5 Institute of Stomatology, Riga Stradins University, Riga, Latvia; 6 Institute of Biomaterials and Bioengineering, Faculty of Natural Sciences and Technology, Riga Technical University, Riga, Latvia

**Keywords:** Cannabidiol (CBD), Gingival mesenchymal stem cells, Levan hydrogels, Local drug delivery

## Abstract

**Introduction:**

The nonpsychoactive cannabinoid cannabidiol (CBD) has shown a wide range of pharmacological effects that are beneficial for wound healing. However, its local delivery is challenged by a very low aqueous solubility.

**Methods:**

In this work, we synthesized hierarchical hydrogels made of the fructan hydrolyzed levan crosslinked with glycerol diglycidyl ether and loaded them with CBD nanoencapsulated within Pluronic^® ^F127 polymeric micelles (25% w/w payload).

**Results:**

Hydrogels showed the typical porous structure (high resolution-scanning electron microscopy) and water uptake capacity up to ~ 1700%. The CBD release kinetics was studied in water (pH 6.8) and phosphate buffered saline (pH 7.4) under sink conditions, at 37°C. An initial burst release stage within the first 2 h of the assay was followed by a more sustained release stage over 72 h. As expected, hydrogels with a lower crosslinking density exhibited faster CBD release in both media. Release data fit the Korsmeyer-Peppas model with a combined mechanism involving diffusion and polymer chain relaxation together with the release of CBD-loaded polymeric micelles. The good compatibility of the hydrogels was initially confirmed in the monocyte-derived human macrophage cell line THP-1 over 72 h. Then, we showed > 70% viability of primary patient-derived gingival mesenchymal stem cells (GMSCs) exposed to hydrolyzed levan solutions, CBD-loaded polymeric micelle suspensions, and the CBD-loaded hydrogels for 28 days. Finally, we conducted preliminary differentiation studies of GMSCs cultured on non-loaded and CBD-loaded hydrolyzed levan hydrogels. Non-loaded hydrogels promote a transient increase in the secretion of the osteogenic marker alkaline phosphatase secretion that peaked at day 7 and declined thereafter, while CBD-loaded ones promote adipogenic differentiation.

**Conclusion:**

Overall, results demonstrate the potential of levan hydrogels as platforms for local drug delivery applications.

**Graphical Abstract:**

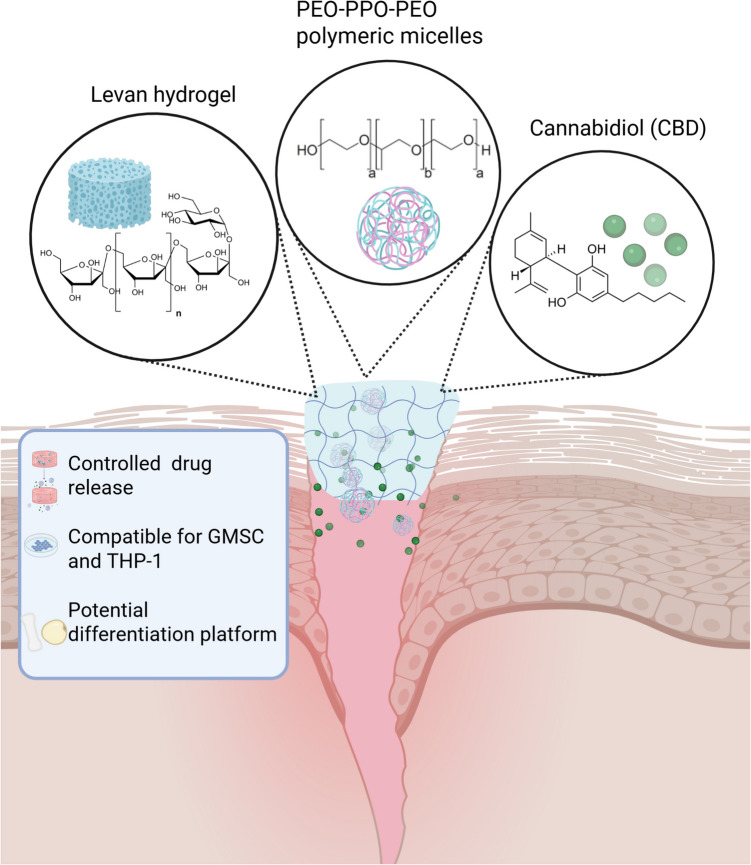

## Introduction

Tissue injury and chronic wounds are disruptions in the continuity of the skin and underlying tissues are a growing global health concern with significant clinical and socioeconomic impact [1, 2]. Approximately 4.4 million people die every year from injuries, with non-fatal cases contributing substantially to long-term disability and reduced quality of life (World Health Organization, 2024).[2] When the healing process is disrupted or fails to progress, acute wounds may evolve into chronic ones.[1],[3, 4] Conventional treatments such as wound dressings, autografts, allografts, or synthetic implants, present several limitations including donor site morbidity, immune rejection, infection risk, and high cost.[3],[5, 6] These challenges underscore the urgent need for new therapeutic strategies.[3, 6]

**Fig. 1:**
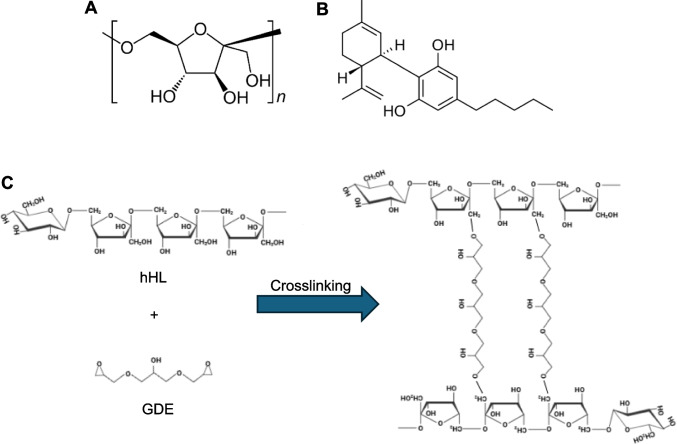
Chemical structures of (**A**) linear levan with beta-2,6 glycosidic linkages and (**B**) cannabidiol (CBD). (**C**) Chemical pathway for the crosslinking of hydrolyzed *Halomonas* levan (hHL) with glycerol diglycidyl ether (GDE).

Hydrogels have emerged as a leading class of biomaterials for tissue engineering and wound healing applications.[5],[7] These three-dimensional, porous, and water-rich hydrophilic polymer networks can retain high water content, mimicking the extracellular matrix of soft tissues.[5, 7,][8] They can be engineered with a range of physicochemical properties by adjusting polymer composition, crosslinking density, and degradation profiles and this tunability allows hydrogels to be tailored for specific tissues or therapeutic applications. Hydrogels can be also engineered as local drug delivery systems [9]. Polysaccharides stand out due to their intrinsic biocompatibility, biodegradability, and chemical versatility [10–13]. Fructans, natural polymers composed primarily of fructose repeating units, have drawn particular interest due to their distinctive architecture and multifunctional properties [14, 15]. They are derived from plants and microorganisms and are known for their ability to form hydrogels, films, and nanoparticles [16]. These features make them promising candidates for biomedical applications that require moisture retention, bioactivity, and localized therapeutic delivery.[10] Levan (Fig. 1), a high-molecular-weight fructan, is especially promising for wound healing applications.[16],[17–21] It is produced through microbial fermentation and is characterized by excellent water solubility, film-forming ability, and elasticity, which allows it to form hydrogels that conform well to wound surfaces and maintain an optimal healing process.[20, 21,][22] In addition to its mechanical functionality, levan is antioxidant, anti-inflammatory, and antibacterial, all activities that could improve wound healing outcomes.[16, 19, 22]

Recent developments in regenerative medicine have revitalized the interest in natural antimicrobial, anti-inflammatory, and antioxidant molecules as therapeutic agents in wound healing [23–26]. Incorporating these natural bioactive molecules into advanced biomaterial platforms such as hydrogels, microparticles, nanoparticles and additional scaffolds, enhances their stability, enables sustained and localized release, and improves therapeutic outcomes while minimizing systemic toxicity. Cannabidiol (CBD, Fig. 1B), a nonpsychoactive phytocannabinoid derived from the *Cannabis sativa* plant, has demonstrated a wide range of pharmacological effects that are beneficial for tissue repair, including anti-inflammatory, antioxidant, analgesic, and pro-angiogenic activities [27–29]. The clinical use of CBD is often jeopardized by its poor aqueous solubility, chemical instability in physiological conditions (e.g., it is photosensitive and prone to oxidation), and extensive hepatic first-pass metabolism after oral administration, which together result in low and inconsistent oral bioavailability [30]. These biopharmaceutical pharmacokinetic drawbacks have driven the development of advanced nano-drug delivery systems designed to improve the aqueous solubility and physicochemical stability, pharmacokinetic profile and therapeutic efficacy of CBD [31]. We previously reported on different types of polymeric nanoparticles for CBD encapsulation and delivery by the ocular and oral routes.[32, 33].

Taking advantage of the ability to produce levan hydrogels by simple crosslinking chemistries and the good encapsulation capacity of the linear poly(ethylene oxide)-*b*-poly(propylene oxide) (PEO-PPO) block copolymer Pluronic F127 for hydrophobic cargos such as the antiretroviral efavirenz with similar physicochemical properties (e.g. melting temperature and intrinsic water solubility) to CBD [34], in this work, we designed and characterized a hierarchical CBD delivery system comprised of CBD-loaded Pluronic^®^ F127 polymeric micelles incorporated into chemically crosslinked hydrolyzed *Halomonas* levan hydrogels for local administration in soft tissue wound healing. The delivery systems are evaluated in terms of physicochemical properties and structure, CBD release kinetics, and preliminary biological performance *in vitro*, including cell compatibility with human monocyte-derived THP-1 macrophages and patient-derived gingival mesenchymal stems cells (GMSCs). Furthermore, the capacity of these hydrogels to support long-term GMSC culture and differentiation into osteogenic and adipogenic lineages is preliminarily explored. Overall results offer new insights into the design of multifunctional, biocompatible scaffolds capable of delivering therapeutic agents and supporting stem cell–mediated tissue regeneration.

## Experimental Section

### Materials

Levan was produced enzymatically using recombinant *Halomonas smyrnensis* levansucrase enzyme (see below). Cannabidiol (CBD, powder, ≥ 98% purity) was supplied from CBDepot (CBDepot s.r.o., Teplice, Czech Republic). Phosphate-buffered saline of pH 7.4 (PBS, Sigma-Aldrich, St. Louis, MO, USA), was used as a buffer in swelling and CBD release studies. Glycerol diglycidyl ether (GDE, Sigma-Aldrich) was used as crosslinker for hydrogel formation. All the reagents were used as supplied.

### Methods

#### Production and Characterization of Hydrolyzed Halomonas Levan. Halomonas Levan (HL)

Was produced enzymatically in a two-step process: (i) Expression and purification of the levansucrase enzyme from *H. smyrnensis* in recombinant *Escherichia coli (E. coli)* and (ii) levan production in a sucrose containing reaction [35]. In brief, *E. coli* BL21 (DE3) cells harboring the pET28_sacB plasmid which contains the *sacB* levansucrase gene were grown in lysogenic broth (LB) medium in an orbital shaker at 37°C and 180 rpm and then induced at the pre-stationary phase of their growth with isopropyl-β-D-thiogalactopyranoside (IPTG, neoFroxx, Einhausen, Germany). After overnight growth at 26°C, cells were collected by centrifugation and lysed with sonication in the cell lysis buffer (20 mM phosphate buffer with 300 mM NaCl, pH 6.6). After centrifugation, the clarified cell lysate was passed through a Ni–NTA His-Bind Superflow affinity column (IMAC; Ni–NTA Superflow, QIAGEN, Hilden, Germany) and active fractions were collected. Then, the pure enzyme was added to the substrate solution containing 0.3M sucrose and 3.5M NaCl and left overnight at 15°C to allow the synthesis of levan. The polymer in the reaction mixture was recovered by precipitation with an equal volume of absolute ethanol and dialyzed in cellulose membrane dialysis tubing with 14,000 Da of typical molecular weight cut-off (Sigma-Aldrich) against water for several days. Finally, the aqueous levan solution was purified by ion exchange chromatography in a Sepharose 6B weak ion column (Sigma-Aldrich) to remove charged impurities. The product was obtained after air-drying at 55°C in a vacuum oven (Wisd Precise Vacuum oven OV-30, witeg Labortechnik GmbH, Wertheim, Germany). Levan was hydrolyzed by microwave-assisted acid hydrolysis in acetic acid (5% w/v) in a house-hold microwave oven (Vestel MW GD-23, Manisa, Turkey) operated at 60% of the maximum power (700W), for up to 3 min [36]. Cold absolute ethanol was added to the microwave-irradiated levan solution, the precipitate was stored at −20°C overnight to enable the precipitation of hydrolyzed *Halomonas* levan﻿ (hHL), the dispersion centrifuged at 8000 rpm for 30 min (Hermle Z36 HK, Gosheim, Germany) and the levan precipitate collected and dried in a vacuum oven (Wisd Precise Vacuum Oven OV-30, witeg Labortechnik GmbH, Wertheim, Germany). The molecular weight distribution of hHL was determined by gel permeation chromatography (GPC) in a Tosoh EcoSEC HLC-8320-RI-UV GPC (Tosoh Bioscience, Tokyo, Japan) equipped with HQ-806M and HQ-807M columns and WYATT HELEOS-II MALS DynaPro DLS detector (Wyatt Technology Corp., Goleta, CA, USA). The molecular weight was 415.5 ± 71.1 kg/mol.

#### Synthesis of Hydrolyzed Levan Hydrogels

hHL hydrogels were prepared in a 24-well plate by crosslinking an aqueous solution of hHL (7% w/v in 0.25 M NaOH, Sigma-Aldrich) with GDE. The hHL solution was magnetically stirred at 300 RPM and GDE was gradually added to achieve crosslinker: hHL volume ratios of 1:25, 1:30, and 1:35. These ratios represent increasing crosslinking densities, with 1:25 indicating the highest level of crosslinking and 1:35 the lowest one. Stirring was allowed room temperature (RT) to ensure homogeneity and the reaction mixtures incubated in an oven at 55°C overnight to complete the crosslinking. Then, hydrogels were carefully extracted from the well plates and washed with ultrapure water (UPW, Milli-Q^®^ Water Purification System, Merck Millipore, Burlington, MA, USA) for three days, with water changes every 5 to 8 h. The washing solution was then replaced with Dulbecco’s Modified Eagle Medium (DMEM, Life Technologies Corp., Carlsbad, CA, USA) to allow the hydrogels to swell in physiological-like conditions for 3 to 5 days. For cell studies, swollen hydrogels were exposed to ultraviolet (UV) light (254 nm) for 30 min per side and cut to fit 96-well plate.

#### Hydrogel Characterization

The mechanical properties of chemically crosslinked hHL hydrogels were evaluated by compression testing using a TA1 Texture Analyzer (Lloyd Instruments Ltd., Bognor Regis, UK). Cylindrical hydrogel samples (2 × 2 cm) were analyzed at hHL-to-crosslinker ratios of 1:25, 1:30, and 1:35. The preload stress was 0.05 N, the preload stress speed 10 mm/min and the compression speed 5 mm/min. The maximum force required to compress the hydrogel, referred to as hardness and expressed in N, was recorded and used as an indicator of mechanical strength and resistance to deformation. A minimum of three replicates was tested per formulation, and results are reported as mean ± S.D.

For water uptake assays, hydrogels were prepared as described above, frozen in liquid nitrogen and freeze-dried (72 h, Labconco Corp., Kansas City, KS, USA). The dry samples (3–8 mg) were placed in 2 mL of UPW (pH 6.8) or PBS (pH 7.4) at 37°C. At 1, 3, 24, and 72 h, samples were blotted gently with wipe paper to eliminate excess liquid from the surface, weighed, and returned to fresh aqueous solution. The water uptake was calculated using Eq. 1

1 $$\text{Water uptake }\left({\%}\right)= \frac{{\mathrm{Q}}_{s}-{Q}_{i}}{{Q}_{i}}*100$$

where Q_i_ represents the mass of the hydrogel in its initial state, and Q_s_ is the mass of the hydrogel in its swollen state.

To gain further information on the hydrogel structure before and after the loading of the CBD-loaded polymeric micelles (see below), we utilized high resolution-scanning electron microscopy (HR-SEM). Freeze-dried samples were carefully sliced in the middle using a scalpel (2 × 2 mm) and then affixed, with one side against the conductive double-sided carbon tape SPI (SPI Suppliers, Structure Probe Inc., West Chester, PA, USA). The samples were visualized at 4 kV in a Zeiss Ultra-Plus Zeiss Ultra-Plus FEG-SEM (Carl Zeiss SMT GmbH, Oberkochen, Germany).

To characterize the hydrogel structure, samples swollen in UPW or in CBD-loaded polymeric micelle solutions, were cut into small square slices (2 × 2 mm) and positioned on the water-containing sample holder of an environmental scanning electron microscopy (E-SEM, FEI E-SEM Quanta 200, Hillsboro, OR, USA). The chamber pressure was gradually reduced from 800 to 330 Pa, while maintaining the sample stage at 3°C. Imaging was performed at an accelerating voltage of 10 kV, and micrographs were captured at multiple pressure points to monitor morphological changes in the hydrogel network throughout the dehydration process.

#### Preparation of and Characterization of CBD-loaded Polymeric Micelles

Pluronic^®^ F127 was purified by dialysis (regenerated cellulose dialysis membranes, molecular weight cut-off of 3500 g mol^−1^, Cellu-Sup T1 nominal flat width of 46 mm, diameter of 29.3 mm, and volume/length ratio of 6.74 mL cm^−1^; Membrane Filtration Products, Inc., Seguin, TX, USA) against UPW over 72 h with frequent water exchanges, freeze-dried and stored at −24°C until use. To prepare 10% w/w Pluronic^®^ F127 polymeric micelles, the copolymer (1.0 g) was dissolved in UPW (9.0 g) and magnetically stirred (300 RPM) overnight at RT. CBD (243 mg) was added to the micellar system resulting in a final CBD loading of 25% w/w (based on the dry weight). The mixture was magnetically stirred (300 RPM) for 24 h in the dark to allow the CBD encapsulation. The resulting CBD-loaded polymeric micelle suspensions contained 25% w/w CBD with respect to the copolymer, were completely transparent to the naked eye, and were stored at 4°C and protected from light until use.

The size (hydrodynamic diameter, D_h_​) and polydispersity index (PDI, an estimation of the size distribution) of CBD-loaded Pluronic^®^F127 polymeric micelles were measured by dynamic light scattering (DLS, Zetasizer Nano-ZS, Malvern Instruments, Malvern, UK) with a 4 mW He–Ne laser (λ = 633 nm), a digital correlator ZEN3600 and Non-Invasive Back Scatter (NIBS^®^) technology at a scattering angle of 173° to the incident beam, at RT. Each measurement was performed in triplicate and results are presented as Mean ± S.D. of at least three independent samples.

#### Incorporation of CBD-loaded Polymeric Micelles into Hydrolyzed Levan Hydrogels

CBD-loaded Pluronic^®^ F127 polymeric micelles were incorporated into hHL hydrogels by the uptake method [37, 38]. For this, weighed freeze-dried hHL hydrogel samples were immersed in a volume of 25% w/w CBD-loaded Pluronic^®^ F127 micellar suspension calibrated to match the maximal uptake capacity of hHL hydrogels, so all the suspension is up-taken. This enabled passive uptake and full rehydration over a 24-h incubation at 37°C. This step facilitated both the structural restoration of the hydrogel and efficient entrapment of CBD-loaded Pluronic^®^ F127 micelles within its polymeric network. The final formulation of CBD-loaded hHL hydrogels was intended to retain the original CBD concentration of the micelle suspension, aiming for accurate dosing and uniform distribution throughout the hydrogel matrix.

#### CBD Release *In Vitro*

For release experiments *in vitro*, CBD-loaded hydrogels were prepared as previously described. Briefly, freeze-dried hHL hydrogels were sectioned into 1.5 mg samples and were rehydrated by incubation with 50 µL of a CBD-loaded Pluronic^®^ F127 micellar suspension (prepared by the dilution of 25% w/w loaded polymeric micelles in culture media to reach a final CBD concentration of 0.00314% w/v) for 24 h, at 37°C [39]. Each loaded hydrogel was transferred into 1.5 mL of UPW (pH 6.8) or PBS (pH 7.4), at 37°C. At 5, 10, and 30 min, and 1, 2, 24, 48, and 96 h, the entire release medium was collected and replaced with fresh medium to ensure sink conditions. The CBD concentration was determined using a modified Beam test [33], for which each sample was mixed with a 5% w/v KOH (Bio-Lab Ltd., Jerusalem, Israel) solution in absolute ethanol (Gadot, Netanya, Israel). Under basic conditions CBD oxidizes to monomeric and dimeric hydroxyquinones, resulting in color change to a violet solution with characteristic absorbance at 330 nm. Absorbance was measured (Multiskan GO, Thermo Fisher Scientific Oy, Vantaa, Finland) and interpolated in a calibration curve of CBD in ethanol in the 0.00003–0.0005% w/v range (R^2^ = 0.9985). The cumulative CBD release (%) was calculated for each time point and data were fitted to different release models using the DDSolver add-in software [40].

#### Preliminary Biological Evaluation

The preliminary biological studies were carried out in human monocyte-derived macrophages and patient-derived GMSCs.

##### Human macrophage compatibility

As a preliminary stage, the compatibility hHL solutions was evaluated using the human monocyte-derived THP-1 macrophage cell line (ATCC^®^ TIB-202™, American Type Culture Collection, Manassas, VA, USA), kindly donated by Prof. David (Dedi) Meiri (Faculty of Biology, Technion – Israel Institute of Technology). THP-1 cells were cultured in RPMI-1640 medium (Life Technologies Corp., Carlsbad, CA, USA) supplemented with L-glutamine, 10% fetal bovine serum (FBS, Sigma-Aldrich), and penicillin/streptomycin (a commercial mixture of 100 U/mL de penicillin + 100 μg/mL streptomycin per 500 mL medium, Sigma-Aldrich), and maintained at 37°C in a humidified incubator with 5% CO_2_. THP-1 monocytes were initially differentiated into macrophages. For this, THP-1 cells were cultured in 96-well plates (20 × 10^3^ cells/well) and phorbol 12-myristate 13-acetate (PMA, Sigma-Aldrich) was added at 50 ng/mL for 24 h in a serum-free-RPMI-1640 medium and allowed to attach to the surface at 37°C and 5% CO_2_. Cells were seeded in 96-well plates at a density of 1 × 10^4^ cells per well and allowed to attach for 24 h. Then, the medium was replaced with fresh RPMI medium containing hHL concentrations ranging from 0.01% to 0.5% w/v. After 24, 48 and 72 h, the medium was removed, and cell viability was assessed via 3-(4,5 dimethylthiazol-2-yl)−2,5-diphenyltetrazolium bromide (MTT) assay. Each well received 100 μL of fresh RPMI medium and 25 μL of sterile MTT solution (5 mg/mL, Sigma-Aldrich). Following a 2-h incubation at 37°C and 5% CO_2_, the resulting formazan crystals were dissolved in 100 μL of dimethyl sulfoxide (DMSO, Sigma-Aldrich), and the absorbance was measured at 530 nm with a reference wavelength of 670 nm using a plate-reader spectrophotometer (Multiskan GO, Thermo Fisher Scientific Oy). Untreated cells were used as controls and considered 100% viable.

##### Human Gingival Mesenchymal Stem Cell Compatibility

The cell viability of hHL solutions and CBD-loaded polymeric micelles was evaluated in human patient-derived GMSCs isolated from patients at the Institute of Stomatology of the Riga Stradins University (Riga, Latvia), characterized prior any further experiments according to the ethical permission Nr.6–1/12/47 of the Research Ethics Committee of Riga Stradins University. For this, GMSCs were cultured in DMEM supplemented with 10% heat-inactivated FBS, and 5 mL of penicillin/streptomycin (a commercial mixture of 100 U/mL de penicillin + 100 μg/mL streptomycin per 500 mL medium) and maintained at 37°C in a humidified, 5% CO_2_ atmosphere. Harvesting was performed using a 0.25% w/v trypsin–EDTA solution, with the culture medium replaced every 2 to 3 days. All experiments were performed using GMSCs between passages 3 and 6. GMSCs were grown in 96-well plates at a density of 1 × 10^4^ cells per well and for 24 h with DMEM and allowed to attach to the surface at 37°C and 5% CO_2_. Then, medium was replaced by fresh DMEM medium and different volumes of a 5% w/v hHL stock solution in PBS were added to obtain final hHL concentrations of 0.05%–1% w/v. Stock levan solutions (5% w/v) in PBS were sterilized by filtration (sterile 0.22 μm syringe filters, Merck Millipore Ltd.) under a biological hood. After 72 h incubation, the medium was removed, and fresh DMEM medium (100 μL) with sterile MTT (25 μL, 5 mg/mL) was added. After 2 h incubation at 37°C and 5% CO_2_, formazan crystals were dissolved in 100 μL of DMSO and the absorbance was measured at 530 nm (with reference to the absorbance at 670 nm) in a spectrophotometer. Control cells  treated only with medium were considered 100% viable.

To assess the GMSC compatibility of 25% w/w CBD-loaded polymeric micelles, diluted in DMEM to final CBD concentrations of 0–12 μM, and applied to GMSCs cultured in 96-well plates (1 × 10^4^ cells/well) for 24, 48, and 72 h, as previously reported to support cell viability at this concentration range [41]. Cell viability was measured by the MTT assay as described above. Control cells were considered 100% viable. All the cell experiments were performed in triplicates and results are expressed as Mean ± S.D. 

Once the GMSC compatibility of hHL solutions and CBD-loaded polymeric micelles was assessed, we studied the compatibility of non-loaded hHL hydrogels in the human monocyte-derived THP-1 and GMSCs. Both cell types were cultured on underneath or on top of pre-cut hydrogel disks (thickness of 3 mm; approx. volume of 100 µL) placed in 96-well plates at a density of 1 × 10^4^ cells per well. To each well, 100 μL of complete culture medium (DMEM for GMSCs and RPMI-1640 for THP-1) was added, and the cells were incubated at 37°C in a humidified 5% CO_2_ atmosphere. For THP-1 cells, the viability was assessed after 72 h, while for GMSCs, the viability was evaluated at 7, 14, 21, and 28 days, with medium changes every 2–3 days. At each designated time point, 20 μL of a freshly prepared solution containing (3-(4,5-dimethylthiazol-2-yl)−5-(3-carboxymethoxyphenyl)−2-(4-sulfophenyl)−2H-tetrazolium) (MTS, 2 mg/mL in PBS, Promega, Madison, WI, USA) and phenazine methosulfate (PMS, 0.92 mg/mL in PBS, Sigma-Aldrich) was added to each well. The MTS and PMS solutions were combined in a 20:1 volume ratio immediately before use. Absorbance of the resulting formazan product was measured at 490 nm. Wells containing cells cultured without hydrogels served as controls and were considered 100% viable.

##### Differentiation of GMSCs *In Vitro*

GMSCs were seeded at a density of 1 × 10^4^ cells/well on top of either non-loaded or CBD-loaded hHL hydrogels in 96-well tissue culture plates. Cultures were maintained for up to 28 days under standard conditions (37°C, 5% CO_2_), with regular culture medium changes every 2–3 days. On days 7, 14, 21, and 28, the culture media were collected from each well and stored at − 20°C until analysis. Differentiation markers were quantified using commercial enzyme-linked immunosorbent assay (ELISA) kits. Human alkaline phosphatase (ALP) as an early osteogenic marker was measured using an ALP ELISA kit (Abcam, Cambridge, MA, USA). Adiponectin, a marker for adipogenesis, was measured using an Adiponectin ELISA kit (Abcam). Assays were performed according to the manufacturer’s instructions in triplicate and the results expressed as Mean ± S.D.

### Statistical Analysis

All measurements, unless otherwise noted, were carried out in triplicate. Results are presented as the mean ± S.D. Statistical analysis was performed by one-way analysis of variance (ANOVA) followed by Tukey's post-hoc test using ﻿GraphPad Prism software version 8.0.1 (GraphPad Software, San Diego, CA, USA). All statistical tests were two-sided, and significance levels are indicated as follows: * for *p *< 0.05, ** for* p* < 0.01; *** for *p* < 0.001 and **** for *p* < 0.0001.

## Results and Discussion

### The Rationale

The therapeutic potential of lipophilic compounds like CBD is often hindered by challenges such as poor aqueous solubility and chemical instability, particularly under physiological conditions. To overcome these limitations, nanocarrier systems have emerged as effective vehicles for enhancing solubility, protecting sensitive molecules, and enabling targeted delivery. Polymeric micelles are self-assembled nanostructures formed by the aggregation of amphiphilic block and graft copolymers above the critical micellar concentration, and they display hydrophilic and hydrophobic domains. The most classical polymeric micelles are formed by amphiphilic diblocks and triblocks that display a typical core–shell structure in which the hydrophobic core is used to encapsulate hydrophobic cargos [42]. Owing to their encapsulation capacity and biocompatibility, linear and branched poly(ethylene oxide)-*b*-poly(propylene oxide) (PEO-PPO) block copolymers are among the most extensively investigated amphiphiles to produce polymeric micelles [43]. Pluronic^®^ F127, a linear PEO-PPO-PEO triblock copolymer with a molecular weight of 12.6 kg mol^−1^ and 70% w/w PEO content, has demonstrated excellent performance for the encapsulation and delivery of different hydrophobic cargos and has been approved by the US-Food and Drug Administration as pharmaceutical, being commercially available in pharmaceutical grade [44]. We anticipated that nanoencapsulation of CBD within polymeric micelles followed by their incorporation into a levan-based hydrogel matrix will enable a more controlled release kinetics for potential application in soft tissue wound healing.

### Preparation and Characterization of CBD-loaded Hydrolyzed Levan Hydrogels

Levan from *H. smyrnensis* is a high molecular weight, adhesive polysaccharide that tends to self-assemble into compact nanospheres in water due to intramolecular hydrogen bonding [25]. While structurally stable, this compactness limits the accessibility of functional groups, reducing the efficiency of chemical modification. To improve reactivity and enable uniform hydrogel formation, levan was hydrolyzed to decrease its molecular weight and increase the availability of reactive termini.

**Fig. 2:**
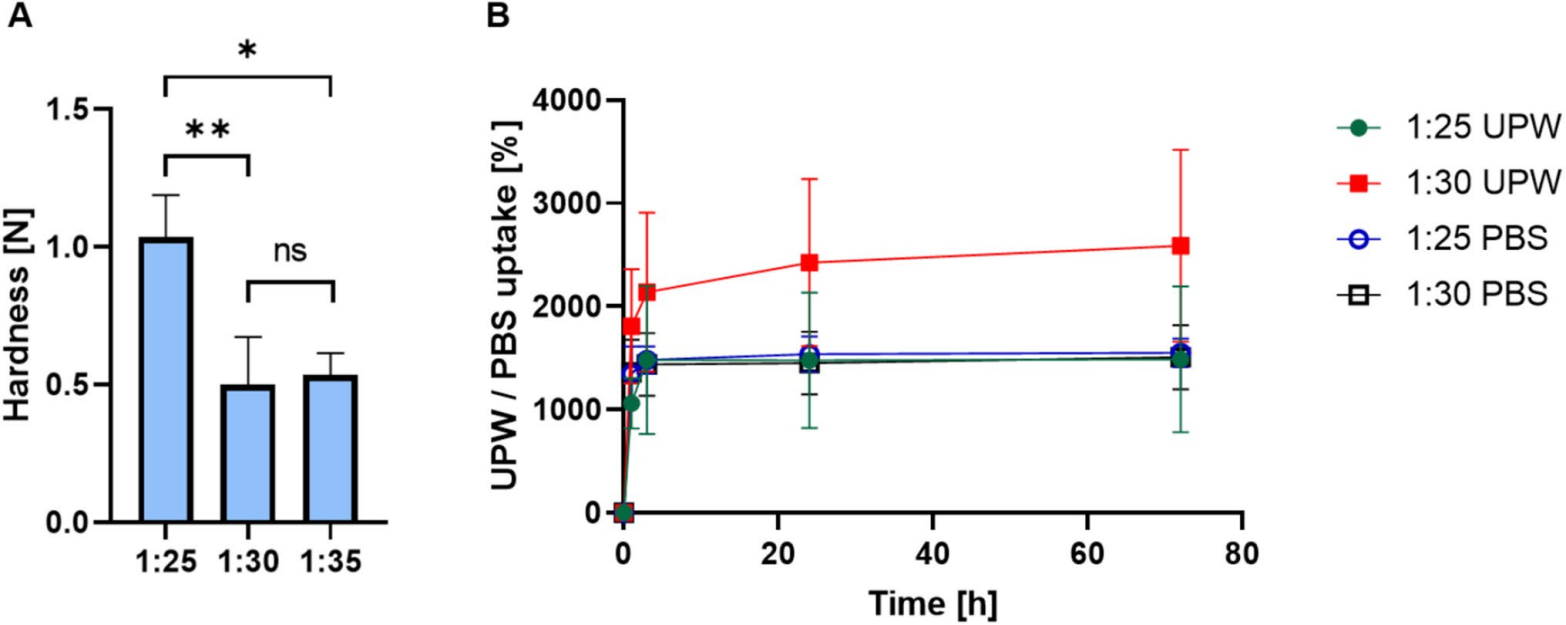
(**A**) Hardness of different hHL hydrogels, as measured by texture analysis. Statistical analysis was performed using one-way ANOVA followed by Tukey’s post-hoc test (GraphPad). Statistically significant differences are indicated by * *p* < 0.05 and ** *p* < 0.01; ns denotes no statistical significance. (**B**) Water uptake kinetics of hHL in UPW and PBS over 72 h, at 37°C. All the results are expressed as mean ± S.D. (*n* = 3).

Previous studies have shown that levan-based hydrogels possess excellent biocompatibility, moisture retention, and structural properties suitable for biomedical use [28]. Building on these findings, we aimed to develop and optimize a levan-based hydrogel system capable of efficient drug incorporation and sustained release, while also assessing its biological compatibility using cell types that are involved in wound healing pathways. To achieve this, hHL was crosslinked using GDE under alkaline conditions. GDE was selected due to its bifunctional epoxide groups, which are known to react efficiently with hydroxyl groups on polysaccharides, forming stable ether linkages (Fig. 1C). Different ratios between both components were screened to produce hydrogels with different crosslinking density and characterized. To assess the effect of crosslinker concentration on the mechanical properties of the hydrogels, we prepared hydrogels at crosslinker:hHL ratios of 1:25, 1:30, and 1:35, and evaluated their mechanical strength in a texture analyzer. Samples with higher crosslinker composition (1:25 levan:crosslinker) exhibited greater hardness of 1.03 ± 0.15 N than the 1:30 and 1:35 counterparts (0.5–0.78 N), indicating enhanced crosslink density and mechanical integrity at higher crosslinker concentrations (Fig. 2A). Since no statistically significant differences were observed between the 1:30 and 1:35 hydrogels, in advance, we only used 1:25 and 1:30 systems.

This relationship between mechanical properties and crosslinker concentration is well-established, as higher crosslinking densities enhance the rigidity and reduce the mobility of polymer chains within the hydrogel matrix and increase the hardness [45]. These hardness values are in line with those shown for other hydrogels mimicking the extracellular matrix of soft tissues [46]. The water uptake capacity of the freeze-dried levan hydrogels was evaluated in UPW and PBS, at 37°C. A fast water uptake of approximately 1100–1300% was observed for all the samples during the first three hours of the experiment owing to the very hydrophilic nature of levan and the crosslinker which bears a free hydroxyl group, after which the curves plateaued and approached the water uptake equilibrium by ~ 24 h (Fig. 2B). In addition, hydrogels showed structural integrity, and no erosion or disintegration were recorded, confirming the efficient chemical crosslinking achieved with GDE. After 72 h, the hydrogels absorbed approximately 1500% of their weight. There were no statistically significant differences between the 1:25 and 1:30 samples in the PBS and the 1:25 in UPW. In contrast, the 1:30 UPW sample absorbed significantly more UPW than the others, reaching above 2000% of its weight (Fig. 2B). Similar water uptake extents at equilibrium suggest that the moderate change in the crosslinker:levan ratio (and the crosslink density) between 1:25 and 1:30 samples does not substantially alter the overall water-holding capacity of the hydrogel under these conditions. Accordingly, the difference in ionic strength between PBS and UPW had a limited effect on the overall water uptake, likely due to the non-ionic nature of the fructan backbone and the crosslinker. However, the significantly higher absorption observed in the 1:30 UPW sample suggests that under specific conditions, lower crosslinking density combined with minimal ionic content may enhance water uptake. Overall, the ability of these hydrogels to retain high water content aligns with the properties of extracellular matrix-mimicking polysaccharide-based materials, supporting tissue hydration, nutrient diffusion, and the maintenance of a moist environment favorable for wound healing.

The loading of lipophilic cargos within hydrogels requires the utilization of advanced strategies that overcome the inherent incompatibility of both components [47]. In this work, we nanoencapsulated CBD within polymeric micelles of Pluronic^®^ F127 by the simple equilibrium method [48], as previously carried for cargos with similar lipophilicity and melting temperature, two physicochemical features that affect the encapsulation capacity for a given amphiphilic copolymer [34]. For this, we screened the CBD loading capacity of 10% w/w Pluronic^®^ F127 polymeric micelles, as we usually do with any new cargo that we aim to nanoencapsulate, as assessed their size by DLS and physical stability over time by the appearance of a precipitate visible to the naked eye. The encapsulation capacity of these polymeric micelles for this cannabinoid was slightly higher than 25% w/w, reaching up to 30% w/w. Nanoformulations were initially transparent to the naked eye, and CBD-loaded polymeric micelles displayed size in the nanometer range and small PDI values [49]; e.g., a 27% w/w CBD-loaded nanoformulation showed D_h_ by intensity and number of 46 ± 1 nm and 23 ± 2 nm, respectively. However, polymeric micelles containing > 25% w/w CBD were physically unstable, and the cargo precipitated fast. Conversely, a payload of 25% w/w rendered transparent micellar dispersions with a D_h_ of 34 ± 1 nm by intensity and 33 ± 6 nm by number, relatively small PDI values, as measured by DLS (Table I), and remained physical stable for relatively long time. Thus, we decided to utilize this system for loading CBD into the hydrolyzed levan hydrogels.

**Table I:** Characterization of CBD-loaded Pluronic^®^ F127 polymeric micelles suspensions before loading and after release from hHL hydrogels as measured by DLS, at 25°C

CBD-loaded Pluronic® F127 polymeric micelles suspensions	D_h_ by Relative Intensity ± S.D. (nm)	D_h_ by Relative Number ± S.D. (nm)	PDI ± S.D
Before loading into hHL hydrogels	34 ± 1	33 ± 6	0.234 ± 0.006
After release from hHL hydrogels	49 ± 6	42 ± 8	0.139 ± 0.035

**Fig. 3:**
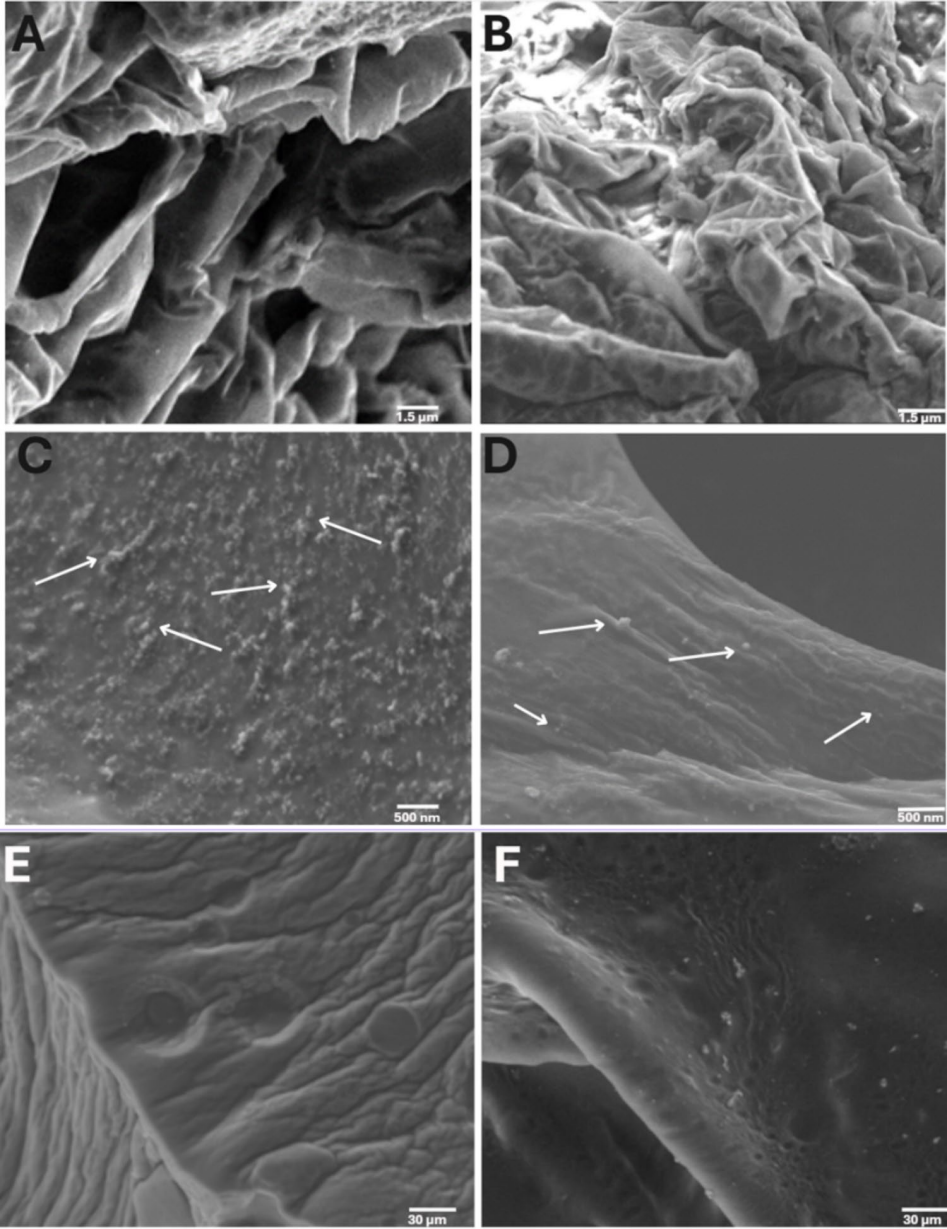
Microstructural characterization of non-loaded and CBD-loaded hHL hydrogels. (**A**-**D**) HR-SEM micrographs of (**A**) non-loaded 1:25, (**B**) non-loaded 1:30, (**C**) CBD-loaded 1:25 and (**D**) CBD-loaded 1:30 hydrogels. (**E**,**F**) E-SEM micrographs of (**E**) non-loaded and (**F**) CBD-loaded 1:25 hydrogels. Arrows point out nanostructures consistent with CBD-loaded polymeric micelles.

A key feature of hydrogels designed for drug delivery applications is high and interconnected porosity which ensures the retention of relatively large water amounts, and the sustained release of the cargo. To incorporate CBD-loaded polymeric micelles into the crosslinked hydrogels, we utilized the swelling method in which the hydrogels are first freeze-dried and then re-swollen in an identical volume of liquid containing the drug-loaded nanoparticles [50]. On one hand, this procedure ensures that all the CBD nanoformulation is incorporated into the hydrogel. On the other, it does not enable the loading of greater CBD payloads. One possible way to overcome the limitation of the swelling volume would be to utilize a more concentrated micellar system which allows the loading of greater CBD amounts in the same volume. In this regard, the reverse thermal gelation of Pluronic^®^ F127 systems upon heating should be considered (e.g., a 17% w/v Pluronic^®^ F127 micellar dispersion gels at approximately 23–25°C)[51] and the process carried out at a temperature that does not induce gelation of the micellar dispersion to ensure the homogeneous swelling of the hydrogel and distribution of the CBD-loaded polymeric micelles in it. The thermal gelation of the CBD-loaded polymeric micelles within the crosslinked hydrogel could be exploited to favor their more efficient retention within the hydrogel matrix and to achieve an even better control of the release kinetics for longer times. These studies were beyond the scope of this first work and are possibilities towards the design of more complex delivery systems. The structure of the hydrogels before and after incorporation of CBD-loaded polymeric micelles was analyzed by HR-SEM that revealed the porous internal architecture of these hHL hydrogels under dry state, confirming the successful formation of a 3D network. In the dry state, HR-SEM of non-loaded hydrogels showed a porous structure (Fig. 3A,B). Importantly, the microstructure hydrogels prepared with different crosslinker ratios were similar, which is consistent with the comparable water uptake behavior described above (Fig. 2B). In the CBD-loaded hydrogels, additional nanostructures that were not visible in non-loaded samples were discernible on the interior surfaces of the pores (Fig. 3C,D) and most likely correspond to CBD-loaded Pluronic^®^ F127 polymeric micelles that are homogenously distributed within the hydrogel matrix upon loading without disrupting the polysaccharide network.

E-SEM was performed on hydrated samples under partial vacuum and results showed the typical surface of highly hydrated chemically crosslinked hydrogels with some signs of porosity[52] and no major changes upon incorporation of CBD-loaded polymeric micelles into the network (Fig. 3E,F). Together, HR-SEM and E-SEM analyses confirm that the synthesized hHL hydrogels form a stable, porous system.

### CBD Release *In Vitro*

The nanoencapsulation of CBD within Pluronic^®^ F127 polymeric micelles was aimed at overcoming its poor water solubility and thus enabling its homogenous loading into the hydrophilic levan matrix, and at better controlling its release with respect to cargos directly loaded into the hydrogel.

**Fig. 4:**
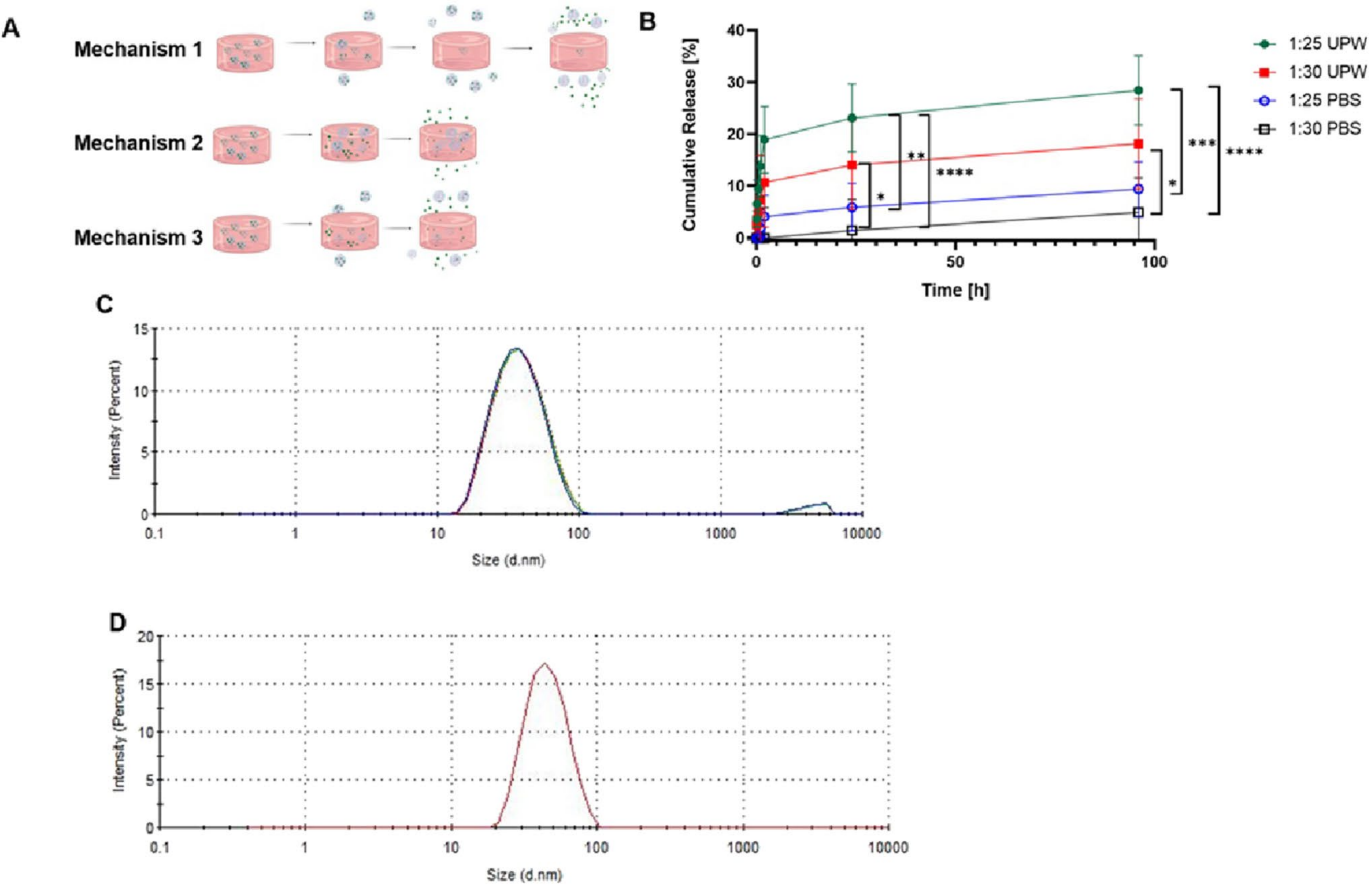
Characterization of the CBD release *in vitro.* (**A**) Possible CBD release mechanisms. (**B**) Cumulative release of CBD from hHL hydrogels in UPW and PBS, at 37°C under sink conditions. Results are expressed as mean ± S.D (*n* = 3). The curves represent the fitting of the release data to the Korsemeyer-Peppas model. Statistical analysis was performed using one-way ANOVA followed by Tukey’s post- hoc test (GraphPad). Statistically significant differences are indicated by * *p* < 0.05; ** *p* < 0.01; *** *p* < 0.001 and **** *p* < 0.0001. (**C***,***D**) Size and size distribution of 25% w/w CBD-loaded Pluronic^®^ F127 polymeric micelles (**C**) in loading medium and (**D**) in release medium after 96 h, as measured by DLS at 37°C (*n* = 3).

In these hierarchical systems of drug-loaded self-assembled polymeric nanocarriers incorporated into a hydrophilic swollen hydrogel matrix, three potential mechanisms could lead to the release of CBD from the hydrogel system to the release medium (Fig. 4A):

(i) Mechanism 1, a one-step mechanism comprised of the direct release of intact CBD-loaded polymeric micelles from the hydrogel to the release medium; once outside the hydrogels, CBD-loaded polymeric micelles can release CBD as a free molecule. In this mechanism, polymeric micelles are not retained within the hydrogel.
(ii) Mechanism 2, a two-step mechanism comprising the release of free CBD molecules from the polymeric micelles to the water within the hydrogel matrix and later diffusion of free CBD out of the hydrogel. In this mechanism, polymeric micelles are retained within the hydrogel.
(iii) Mechanism 3, a combination of Mechanisms 1 and 2.

The release kinetics was assessed in two media, namely UPW (pH 6.8) and PBS (pH 7.4), at 37°C. Even though the ionic strength gap between both media did not affect the water uptake, it could change the intrinsic solubility of CBD and affect the sink conditions and the release. In general, the release profiles from 1:25 and 1:30 systems were biphasic, with an initial moderate burst effect most probably due to the release of CBD-loaded micelles in the outer regions of the hydrogel, followed by a more prolonged slow-release phase (Fig. 4B). After ~ 6–8 h, the release rate markedly declined, and CBD was released in a more sustained manner over the subsequent days. By 96 h, the cumulative CBD release from the hydrogels reached only a fraction of the total loaded dose. Notably, the extent of release depended on both the crosslinking density of the hydrogel and the medium composition. In UPW, the 1:25 hydrogel released approximately 28.5 ± 2.5% of its CBD payload, whereas the 1:30 counterpart approximately 18.1 ± 2.2%. In PBS, the overall release was slower, 9.4 ± 2.1% and 4.9 ± 2.5% for 1:25 and 1:30 hydrogels. This outcome suggests that CBD solubility and the physicochemical interactions between the hydrogel network and the surrounding medium play a more dominant role in release behavior than crosslink density alone. In UPW, the absence of ions may allow more uniform swelling and greater water uptake, even in more crosslinked matrices, facilitating enhanced CBD diffusion. Additionally, the tighter network of the highly crosslinked hydrogel may reduce CBD entrapment and promote faster expulsion under favorable solubility conditions. Conversely, in PBS, the low aqueous solubility of CBD (~ 10 µg/mL) significantly limits its release, regardless of hydrogel crosslink density. Furthermore, the presence of salts in PBS may screen electrostatic interactions within the hydrogel and suppress swelling, especially in the less crosslinked system, thereby further hindering CBD mobility.

These results indicate that a lower crosslinking density and ionic strength environment promote the faster CBD release and would fit the application of these systems in long-term local drug delivery. The inverse relationship between crosslink ratio and release rate is consistent with the notion that a more loosely crosslinked polymer network presents larger mesh sizes and less hindrance to diffusing molecules [53, 54]. To reveal whether CBD-loaded polymeric micelles are released from the hydrogel or not, release aliquots were analyzed by DLS (Table I), which showed the presence of nanoparticles with a size, as expressed by the hydrodynamic diameter, of 49 ± 6 nm, which is consistent with the presence of CBD-loaded polymeric micelles in the release medium. These results strongly suggest that mechanisms 1 and 3 would be responsible for the relatively low release kinetics observed in these hierarchical systems over the 96 h of the assay. The release of CBD-loaded polymeric micelles was expected because the micellar size is substantially smaller than the pore size of the hydrogels, as strongly suggested by HR-SEM analysis. However, these CBD-loaded delivery systems are envisioned to perform in a wound bed for relatively long times and thus, to exert an anti-inflammatory effect and induce the differentiation of stems cells in it.

To gain a better understanding of the release mechanisms, release data were fitted to different models, including zero-order and first-order kinetics, Higuchi, Korsmeyer-Peppas, Hixson-Crowell and Weibull, using the DDSolver software (Table II) [40]. The best fit was the Korsmeyer–Peppas model (R^2^ between 0.92 and 0.96) for all the samples and release conditions), indicating that release is governed by a combination of Fickian diffusion and structural relaxation of the polymer network. In this model, the release exponent *n* is indicative of the mechanism for a thin hydrogel slab (one-dimensional diffusion), *n* ≈ 0.5 corresponds to Fickian diffusion-controlled release, *n* ≈ 1.0 corresponds to case-II transport (polymer relaxation or erosion-controlled release), and intermediate values 0.5 < *n* < 1.0 indicate anomalous (non-Fickian) kinetics transport – a combination of diffusion and polymer relaxation [55]. For our systems, the n values ranged between 0.51 and 0.75 (Table II), indicating that a combination of diffusion and polymer relaxation could take place. Further optimization of the crosslinking density may allow us to tune the release rate based on a specific application.

**Table II:** Fitting of CBD release data *in vitro* to different models

Condition	Release Model	Fitting (R^2^)	Parameters (n,k)	
1:25 Hydrogel in UPW (pH 6.8)	Zero-order	0.85	-	-
	First-order	0.72	-	-
	Higuchi	0.91	-	-
	Korsmeyer-Peppas	0.95	0.65	0.89
	Hixson-Crowell	0.76	-	-
	Weibull	0.88	-	-
1:30 Hydrogel in UPW (pH 6.8)	Zero-order	0.83	-	-
	First-order	0.70	-	-
	Higuchi	0.89	-	-
	Korsmeyer-Peppas	0.92	0.72	0.85
	Hixson-Crowell	0.74	-	-
	Weibull	0.85	-	-
1:25 Hydrogel in PBS (pH 7.4)	Zero-order	0.87	-	-
	First-order	0.75	-	-
	Higuchi	0.93	-	-
	Korsmeyer-Peppas	0.96	0.68	0.9
	Hixson-Crowell	0.78	-	-
	Weibull	0.90	-	-
1:30 Hydrogel in PBS (pH 7.4)	Zero-order	0.84	-	-
	First-order	0.73	-	-
	Higuchi	0.90	-	-
	Korsmeyer-Peppas	0.94	0.7	0.87
	Hixson-Crowell	0.77	-	-
	Weibull	0.86	-	-

### Preliminary Biological Evaluation

#### Human Macrophage Compatibility

**Fig. 5:**
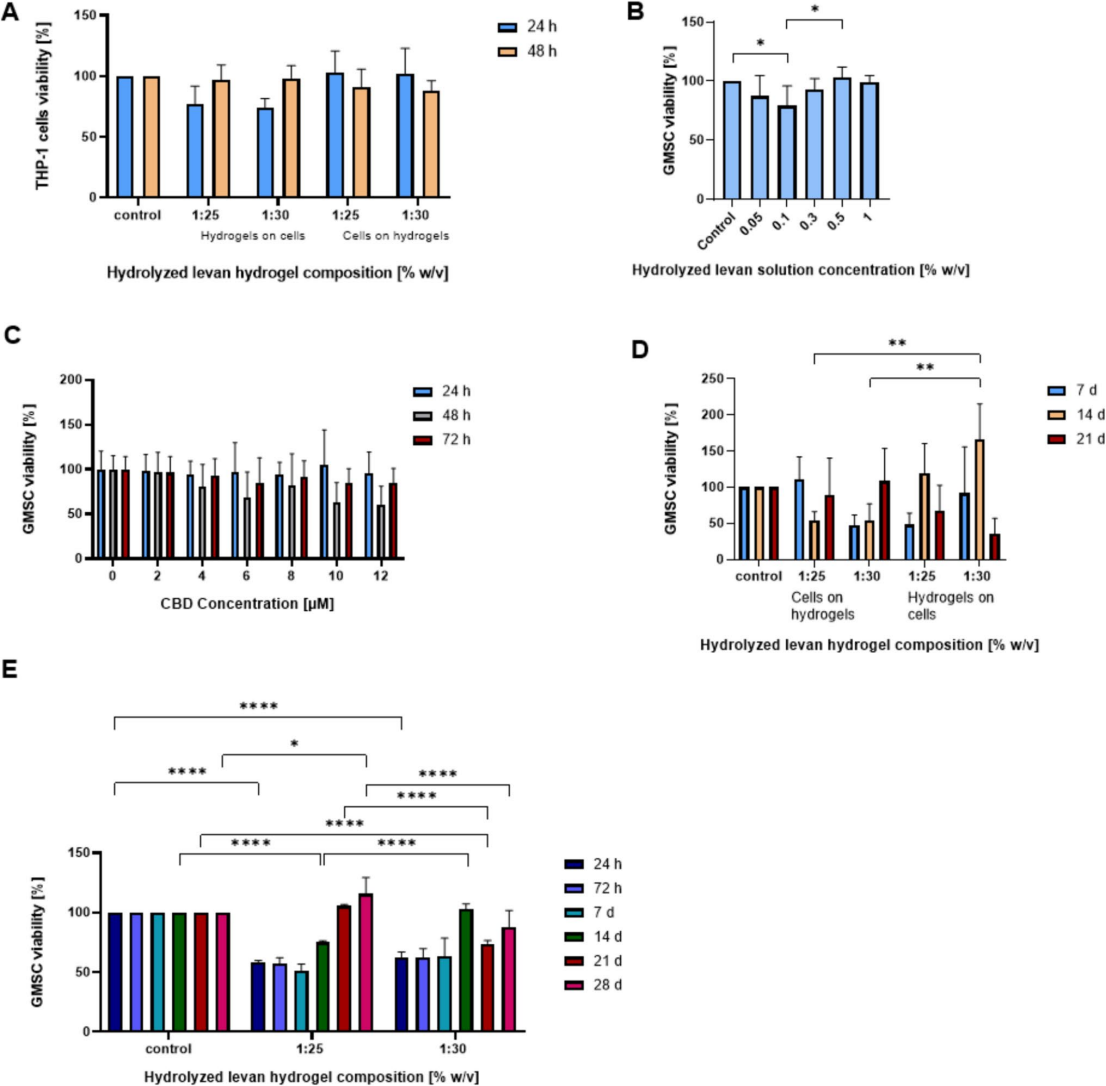
Cell compatibility of hHL solutions and hydrogels, as estimated by MTT and MTS assays. **A** Viability of human monocyte-derived macrophage THP-1 cell line cultured on top of and below hHL hydrogels for 24, 72 h, (B-E) viability of patient-derived GMSCs treated with (**B**) hHL solutions (0.05–1% w/v) for 72 h at 37°C, (**C**) 25% w/w CBD-loaded Pluronic^®^ F127 polymeric micelle suspensions (final CBD concentration of 0–12 µM) for 72 h at 37°C, (**D**) seeded on top and below hHL hydrogels (1:25, 1:30) for 7, 14, 21 days, (**E**) hHL hydrogels (1:25 and 1:30) for 28 days at 37°C. Results are expressed as mean ± S.D. of three independent experiments (*n* = 3). Statistical analysis was conducted using two-way ANOVA, using GraphPad. Statistically significant difference between values (*p < 0.05; ** p < 0.01; *** p < 0.001; **** p < 0.0001), Statistical analysis of GMSC viability in hHL solutions (**B**) was conducted using one-way ANOVA followed by Tukey’s post-hoc test (GraphPad). Statistically significant differences are indicated by * *p* < 0.05; ** p < 0.01 and **** p < 0.0001).

CBD-eluting hHL hydrogels are envisioned for local delivery applications in wound healing. In this context, we initially assessed the cell compatibility of human macrophages and GMSCs cultured on top of non-loaded specimens. This experimental setup mimics better the application in which the hydrogel is implanted in a wound bed, as opposed to cells cultured on tissue culture plates and covered with hydrogel. Macrophages are actively involved in inflammation and wound healing process, and they could be used as a model to optimize the hydrogel properties. In previous work, we reported on the excellent human macrophage cell compatibility of hHL solutions [56]. In a 72-h assay, 1:25 and 1:30 hHL hydrogels showed very good THP-1 macrophage compatibility (viability > 90%), regardless of the crosslinking density and culture setup (underneath or on top) (Fig. 5A), and in good agreement with previous studies conducted with levan solutions in the same cell line.

#### Human Gingival Mesenchymal Stem Cell Compatibility

The long-term aim of this work is to design local drug delivery systems for gingival wound healing. GMSCs are non-hematopoietic adult stem cells isolated from the gingival lamina propria using minimally invasive procedures [57, 58], as opposed to bone marrow MSCs that require invasive isolation procedures [59]. They are abundant and proliferate rapidly, show strong immunomodulatory capacity, multiple differentiation paths and maintain stable properties across passages [60]. In addition, they are more abundant than MSCs in other non-dental and dental tissues. In this framework, the goal of these preliminary *in vitro* experiments was to evaluate the interaction of these hierarchical CBD-loaded delivery systems with stem cells that are available in the wound bed and possible differentiation pathways that are relevant for gingival tissue regeneration.

Initially, we estimated the viability of GMSCs with hHL solutions in the 0.05–1% w/v range by the MTT assay. GMSCs maintained high viability (> 80%) across all tested concentrations of hHL solutions after 72 h of incubation (Fig. 5B). Similarly, 25% w/w CBD-loaded Pluronic^®^ F127 polymeric micelles with final CBD concentrations of 0–12 µM exhibited no major cytotoxic effects, and no statistically significant differences in cell viability were observed across CBD concentrations at any evaluated time point (Fig. 5C). These results were in the same order of magnitude of the cell toxicity of CBD previously reported [41]. At the same time, its nanoencapsulation within polymeric micelles slightly reduced the toxicity, enabling the use of higher effective concentrations without substantial cell death. After establishing that levan solutions are non-cytotoxic, cell viability was evaluated when cells were in direct contact with the solid hydrogel scaffolds.

Cells were cultured underneath and on top of the hydrogels to re-evaluate optimal experimental setup (Fig. 5D). Over a 21-day period, the metabolic activity of GMSCs was monitored using the MTS assay. After 7 days, GMSCs seeded on top of hydrogels with higher crosslinking densities showed viability like the control, while cells on 1:30 hydrogels showed initially low viability. However, the viability in 1:30 samples increased over time, surpassing the control by Day 21. In contrast, 1:25 showed a slight drop by Day 14 but remained above 90% at Day 21. Cells that were cultured underneath the hydrogels had inconsistent trends. In the 1:25 condition, viability was low on Day 7, peaked on Day 14, then dropped below 70% by Day 21. For 1:30, initial viability was good but sharply declined by Day 21, indicating that cells cultured on top of hydrogels provide better support for long-term survival and proliferation. In addition, cells cultured on top of the hydrogels tend to migrate into the matrix, which mimics better the application *in vivo* where the drug delivery system is placed in the wound bed. When GMSCs were cultured on hHL hydrogels (1:25, 1:30) for four weeks, they showed proliferation as indicated by the increase in the metabolic activity recorded by the MTS assay when compared to the same cells cultured on tissue culture plates (Fig. 5E). Initially (Day 7), the cell viability on all hydrogel samples was slightly lower than the control (~ 70% of control), which is not unexpected as cells require some time to adjust to the new 3D matrix environment. However, after this initial period, cell viability steadily increased. By Days 14 and 21, GMSCs viability levels remained high and were like control levels. By Day 28, the metabolic activity equaled was greater than the control (untreated) group for all the different compositions. These results indicate that GMSCs can proliferate in this environment and that the most consistent trends appeared at compositions 1:25 and 1:30 and for these conditions sudden drop of viability was not achieved, making them more favorable environment for prolonged exposure.

### Human Gingival Mesenchymal Stem Cell Differentiation

Following confirmation of cell compatibility, preliminary differentiation assays were conducted to explore whether exposure to non-loaded and CBD-loaded hHL hydrogels could influence lineage-specific responses in GMSCs. GMSC differentiation in different polysaccharide-based hydrogels has been a subject of growing scientific interest [13, 61]. Hydrogel mechanics has been shown to differentially support osteogenic and adipogenic outcomes, with stiffer networks generally favoring osteoblast differentiation, while softer matrices being more permissive to adipogenesis [62, 63]. Beyond mechanical support, certain polysaccharides inherently modulate cellular pathways through their biochemical properties. Among these, levan and other fructans have demonstrated immunomodulatory and pro-regenerative effects, contributing to enhanced cellular responses and supporting differentiation [64]. Levan hydrogels have also shown promise as drug delivery systems [65]. These properties position polysaccharide-based hydrogels as promising platforms for regenerative therapies involving GMSCs.

Several studies have demonstrated that CBD-loaded microparticles and hydrogels can support tissue regeneration, including spinal cord repair, bone defect healing, and MSC recruitment within osteoconductive scaffold [66–69]. CBD has also been reported to promote adipogenic differentiation. Studies indicate that CBD can modulate adipogenesis by activating key transcriptional regulators and restoring differentiation capacity under inflammatory conditions, highlighting its versatile role in influencing MSC fate decisions relevant to regenerative medicine.[71–74].

**Fig. 6:**
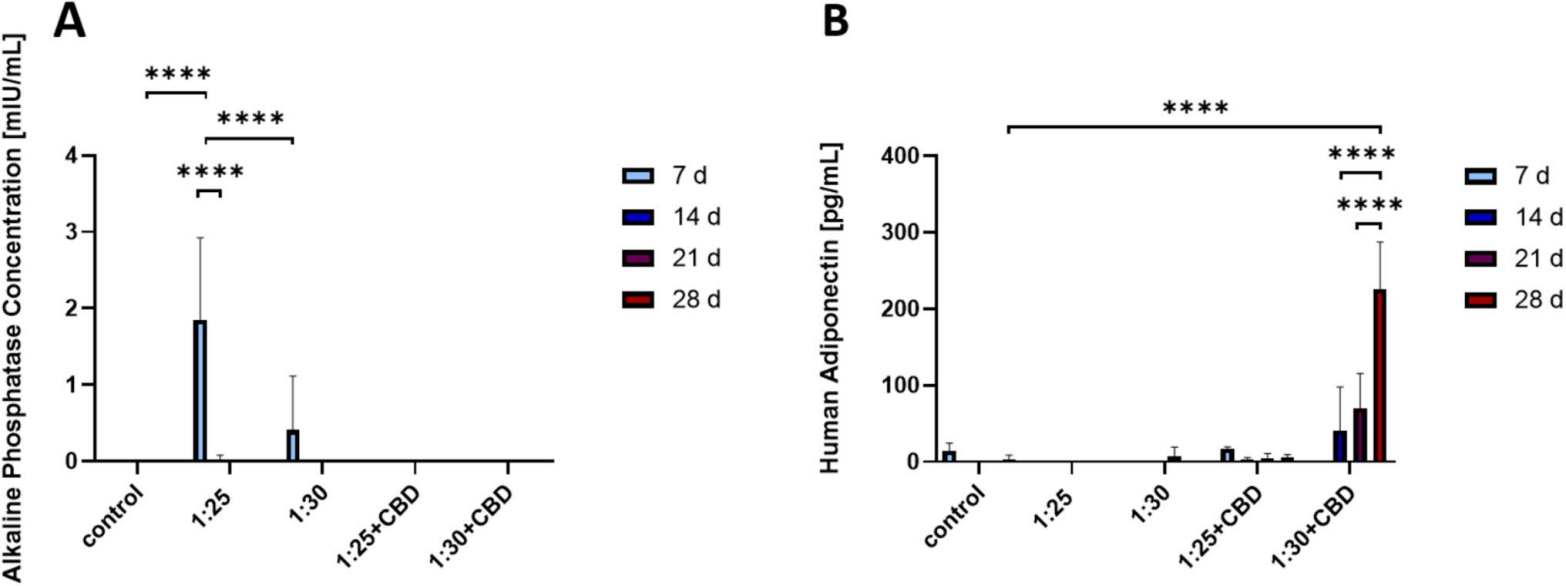
Preliminary differentiation of GMSCs, as measured by ELISA. (**A**) Human alkaline phosphatase secretion and (**B**) human adiponectin levels from GMSCs cultured on hydrogels (non-loaded *vs*. CBD-loaded) over 28 days (ELISA, mean ± S.D., *n* = 3). Statistical analysis was conducted using two-way ANOVA followed by Tukey’s post-hoc test (GraphPad). Statistically significant differences are indicated by **** p < 0.0001.

In this work, we aimed to preliminarily characterize the possible effect of non-loaded and CBD-loaded hHL hydrogels on the differentiation of GMSCs by measuring the secretion of ALP and adiponectin in the culture medium. In the non-loaded levan hydrogels, GMSCs exhibited a transient increase in ALP secretion at Day 7 with a fast decline after it (Fig. 6A). This effect was more pronounced in more crosslinked and harder hydrogels (1:25), a phenomenon that has been already depicted for other hydrogels in which hardness increases osteogenic differentiation [73, 74]. The decline in ALP activity over time could be attributed to cell confluence, a transition toward later differentiation phases, or a lack of additional osteogenic cues in the culture medium and would be consistent with the transient role of ALP as an early marker of osteogenesis, typically peaking during matrix maturation and declining as mineralization progresses and late-stage markers become predominant. In the case of CBD-loaded systems, we expected to observe enhanced osteogenic differentiation, given its role in promoting osteogenesis through signaling pathways [75]. However, no ALP activity was detected in these systems. Several factors may explain this outcome. First, CBD may have preferentially promoted adipogenic over osteogenic differentiation under the given conditions, consistent with reports indicating its ability to stimulate adipocyte maturation and promote intracellular lipid accumulation through modulation of adipogenic signaling pathways [76, 77]. Another potential explanation lies in the altered release kinetics of CBD from the hydrogel matrix, which may have failed to maintain effective concentration over time. Additionally, the timing and duration of CBD exposure likely played a critical role.

Release studies showed an initial burst release of CBD within the first few days, followed by a steep decline. In a 2D culture system with routine medium replacement, this early release may have produced only a transient effect, while subsequent degradation or insufficient sustained release could have limited the effect of this cannabinoid on osteogenic signaling. In the context of adipogenic differentiation, assessed through adiponectin secretion—a well-established marker of mature adipocytes- only the CBD-loaded hHL hydrogels with crosslinking ratios of 1:25 and 1:30 elicited a measurable and progressive increase in adiponectin levels over the 28-day culture period. Among these, the 1:30 formulation demonstrated the most pronounced adipogenic effect. (Fig. 6B). This response may reflect an optimal balance between hydrogel stiffness and bioactive compound availability, as previous studies have shown that intermediate stiffness ranges can enhance adipogenesis by permitting sufficient cell spreading and mechanotransduction. Moreover, the known activation of adipogenic pathways, such as peroxisome proliferator-activated receptor gamma signaling, likely contributed to the observed differentiation. In contrast, no adiponectin secretion was observed in the corresponding non-loaded hydrogel groups. This absence of adipogenic differentiation may be attributed to several factors. First, while polysaccharide-based hydrogels can provide a permissive 3D environment, they may lack the specific biochemical signals required to initiate or sustain adipogenic commitment in the absence of additional inductive cues. Second, the mechanical stiffness of the hydrogels, especially in the more crosslinked 1:25 formulation, could have influenced cell fate, as higher stiffness is known to favor osteogenic rather than adipogenic differentiation. Conversely, the 1:30 hydrogel, despite being softer, may still not have provided sufficient biochemical stimulation or matrix degradation cues to support adipocyte maturation. These findings highlight the importance of integrating bioactive compounds like CBD into hydrogel systems to guide mesenchymal stem cell fate. The observed outcomes point to a synergistic relationship between the mechanical properties of the hHL hydrogel and localized CBD delivery, which together shape lineage-specific differentiation in GMSCs. This reinforces the promise of composite biomaterials for applications in soft tissue regeneration.

## Conclusions

This work investigated the development of a dual-function hHL-based hydrogel system for localized, sustained delivery of CBD. By embedding CBD-loaded Pluronic^®^ F127 micelles into a cytocompatible polysaccharide matrix, we created a hierarchical matrix that both supports cell growth and releases a therapeutic agent over time. The hydrogels exhibited strong uptake properties and mechanical integrity, high cell compatibility with both THP-1 macrophages and GMSCs, and sustained CBD release following a mixed diffusion–relaxation mechanism. Notably, CBD release fits the Korsmeyer-Peppas model, confirming controlled release behavior. Preliminary cell differentiation assays revealed that variations in hHL hydrogels compositions and CBD loading influence stem cell lineage commitment. Specifically, non-loaded hydrogels appeared to promote early osteogenic signals, as shown by increased ALP secretion within the first seven days, while CBD-loaded hydrogels tended to enhance adipogenic marker expression, particularly under lower crosslinking conditions. These early observations point to a complex relationship between the physicochemical properties of these hydrogels and cellular behavior. However, the specific contributions of each parameter-such as CBD concentration, release kinetics, and mechanical stiffness, require further investigation to determine their individual and combined roles in directing stem cell fate. These findings also demonstrate the safety and multifunctionality of CBD-loaded hHL hydrogels as localized drug delivery platforms capable of modulating stem cell responses.

## Data Availability

Data will be available upon request.
